# The Beacon Wiki: Mapping oncological information across the European Union

**DOI:** 10.1186/s12911-025-03015-6

**Published:** 2025-05-19

**Authors:** Veronica Coppini, Giulia Ferraris, Maria Vittoria Ferrari, Dario Monzani, Margherita Dahò, Elisa Fragale, Roberto Grasso, Ricardo Pietrobon, Aline Machiavelli, Lucas Teixeira, Victor Galvão, Gabriella Pravettoni

**Affiliations:** 1 Applied Research Division for Cognitive and Psychological Science, IEO, European Institute of Oncology IRCCS, Milan, Italy; 2 Department of Psychology, Educational Science and Human Movement (SPPEFF), University of Palermo, Palermo, Italy; 3 Department of Oncology and Hemato-Oncology, University of Milan, Milan, Italy; 4 SporeData OÜ Tallin, Tallinn, Estonia

**Keywords:** Oncology, Cancer registries, Metadata, Data repository, Cancer care disparities

## Abstract

**Background:**

Accessing comprehensive oncological data is essential for efficient and quality healthcare delivery and research. However, obstacles, such as data fragmentation and privacy concerns which may hold back progress in this area, exist. The Cancer Care Beacon project addresses these barriers consolidating oncological information across the 27 member states of the European Union (EU) with the goal of creating a Beacon wiki free data online repository.

**Methods:**

The Cancer Care Beacon project involves thorough data collection from various sources, including hospital websites, PubMed, ClinicalTrials.gov, and national health institutions. The main focus of metadata retrieval is placed on descriptive details about data sources, thus warranting compliance with privacy regulations and ethical standards. In addition, manual examination and semi-automated methods are included in the process, enabling a registry of administrative databases, cancer registries, and other relevant databases.

**Results:**

Project findings demonstrate the success in the realisation of a comprehensive repository of oncological data sources across the EU assisting informed decision-making regarding the selection and utilisation of resources. Still, challenges such as limited accessibility and low engagement from database providers persist.

**Conclusion:**

The Beacon Wiki represents a significant step in addressing disparities in oncological data access and advancing cancer care and research in Europe. By providing comprehensive metadata on cancer-related data sources, Beacon Wiki empowers stakeholders and promotes collaboration in cancer care and research. Continuous efforts are needed to enhance data accessibility and engagement from database providers, ultimately improving data-driven decision-making and patient outcomes in the EU.

**Graphical Abstract:**

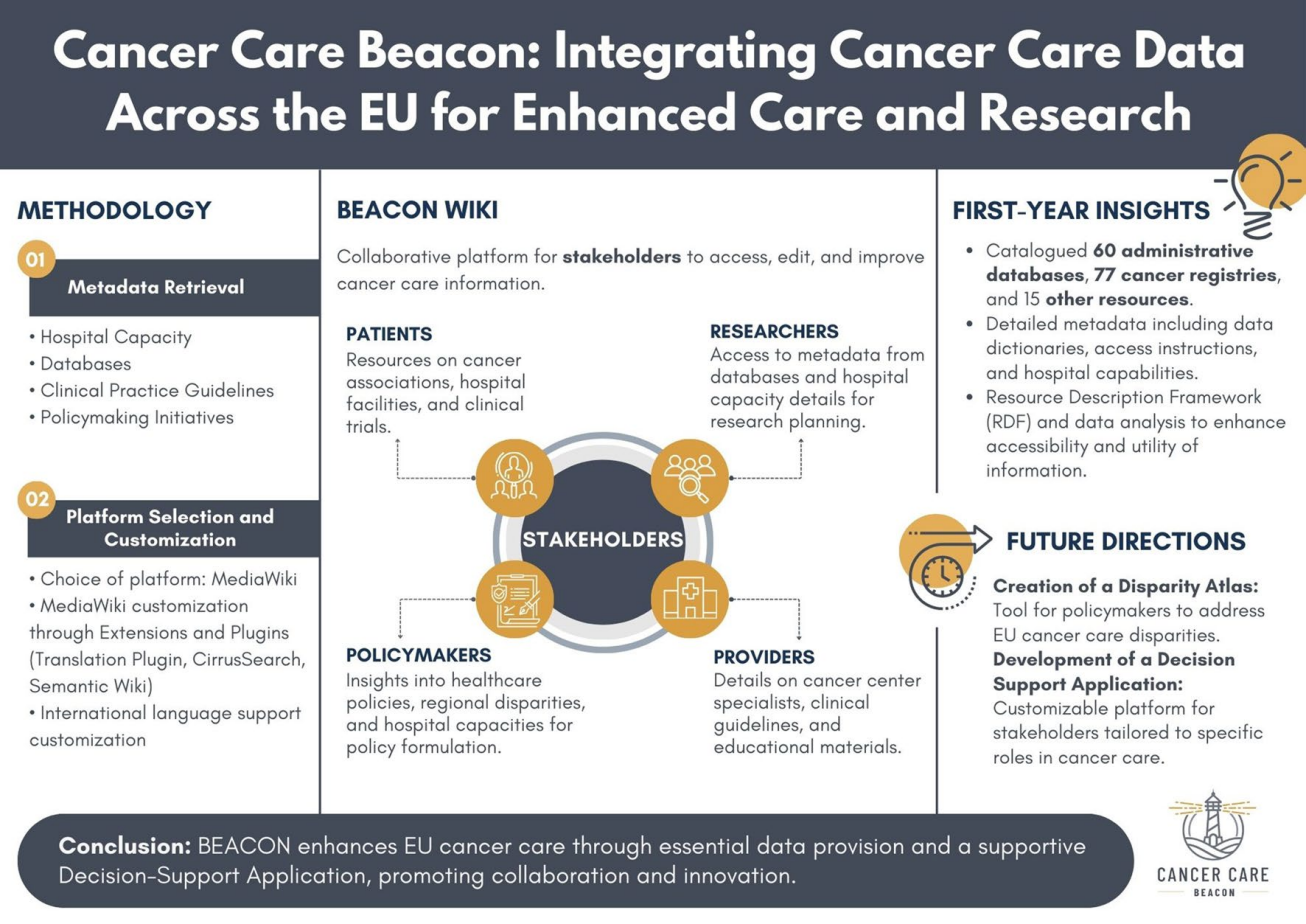

**Supplementary Information:**

The online version contains supplementary material available at 10.1186/s12911-025-03015-6.

## Background

Healthcare systems worldwide rely heavily on access to comprehensive and up-to-date health data for effective and informed decision-making, especially in the realm of oncology. While global institutions like the United States (US) National Institute of Health and Stanford’s AI Medicine and Imaging have embraced open data initiatives, underscoring the necessity of publicly available health research data, the European Union (EU) faces relevant barriers in accessing Electronic Health Data (EHDs), delaying advancements in cancer care and research [1]. Substantial barriers in utilising oncological information, freely and efficiently, arise due to data fragmentation, lack of interoperability, governance complexities, and privacy concerns such as the General Data Protection Regulation (GDPR) [2, 3]. Such barriers have led to detectable disparities in data availability and research output, as displayed by the contrasting utilisation of cancer registry data between the US and the EU [4].

The COVID-19 pandemic has vividly demonstrated the critical importance of readily accessible health data for informed public health measures and crisis response [5]. Within this frame, health system experts stress the importance of resilience, cautioning against oversimplifying it as a one-size-fits-all solution. Resilience emerges from systemic resources and interactions, deeply influenced by power dynamics and relationships within the healthcare system. The pandemic has revealed the need for robust data infrastructure to support resilience-building efforts, allowing for timely and accurate information exchange among stakeholders and, especially, researchers. Additionally, the COVID-19 pandemic has underlined the significance of data transparency and interoperability in enhancing resilience, thus allowing healthcare systems to adapt and respond effectively to evolving challenges [6].

The latest estimates from the International Agency for Research on Cancer (IARC) indicate a significant global burden of cancer, with 20 million new cases and 9.7 million deaths in 2022. Lung cancer remains at the top of the list of new cases, followed by breast, colorectal, and prostate cancers. Estimates suggest a further increase to 35 million new cases by 2050 [7].

A report from the Organisation for Economic Co-operation and Development (OECD), “Beating Cancer inequalities in the EU”, highlights numerous inequalities in cancer incidence and mortality both between and within countries [8]. These disparities are influenced by factors such as educational level and socioeconomic status, with preventable risk factors disproportionately affecting vulnerable populations [9]. Therefore, it is essential to invest in system capabilities, including data collection, analysis, and dissemination mechanisms, in order to foster resilience and access to comprehensive and up-to-date health data [10]. Although initiatives have been launched to address these risk factors, prioritising health equity remains inconsistent across the EU. Indeed, disparities in health equity persist not only between countries but also within regions or cities of the same country. The COVID-19 pandemic has highlighted the structural barriers to health and healthcare and the gaps in public health infrastructure, emphasising the urgency of globally addressing these issues [11]. Thus, fostering collaboration among interconnected social systems, including healthcare providers, researchers, policymakers, and communities, is crucial for navigating complex challenges such as pandemics or health inequalities. By utilising accessible health data and promoting collaboration, healthcare systems can enhance their resilience and capacity to address public health crises effectively [12]. In addition, implementing programs to temporarily improve cancer equity locally or regionally is a good starting point, but it is imperative to develop a public health strategy focussed on alleviating the root causes of health inequities to improve the health conditions and well-being of every citizen and ensure readiness for the addressed public health emergency [11].

In response to these challenges, initiatives such as the development of the Beacon Wiki have emerged to address the need for consolidated and accessible oncological information across the EU. The Beacon Wiki aims to bridge the gap in accessing oncological data by establishing a comprehensive repository mapping various oncological information across the 27 EU member states. This repository includes detailed metadata on cancer hospitals, healthcare databases, cancer registries, reports, clinical practice guidelines, patient associations, and resources pertinent to cancer care. By centralising this information and making it publicly available, the Beacon Wiki seeks to empower stakeholders, including patients, providers, researchers, and policymakers, with critical resources for cancer care and research. Moreover, the collaborative and editable wiki format of Beacon ensures up-to-date and accurate information, facilitating better identification and access to relevant databases for public health research. Additionally, the Beacon Wiki incorporates metadata standards and infrastructure similar to those employed in established metadata services for cancer research [13]. This ensures that the data provided through Beacon adheres to standardised formats and practices, enhancing its usability and interoperability across various research initiatives and institutions. By leveraging metadata principles, the Beacon Wiki streamlines data collection, aggregation, and analysis, further promoting practical solutions for cancer patients and supporting healthcare providers and researchers in their recommendations and investigations, significantly contributing to the advancement of comprehensive cancer care as well as public health research.

This paper discusses whether the creation of a comprehensive, open-access oncological data repository (the Beacon Wiki) could improve data accessibility and support evidence-based decision-making among patients, healthcare providers, researchers, and policymakers across the EU. By exploring this question, we aim to establish the repository’s potential to enhance research, clinical practice, and policymaking within the European oncology landscape.

In the current paper, we provide an overview of the Cancer Care Beacon project’s (BEACON) wiki, outlining the methodology, findings, and implications for cancer care and research in the EU. By addressing challenges in accessing oncological information, BEACON aims to contribute significantly to the advancement of cancer care, reduce disparities, and facilitate data-driven research in Europe.

To illustrate BEACON’s practical utility, in the current paper we included a case study demonstrating how the platform can empower patients in making informed healthcare decisions, highlighting its transformative potential in clinical settings.

## Construction and content

The Beacon project represents a committed attempt aimed at providing a comprehensive repository of metadata concerning various sources of cancer-related information. This effort was specifically tailored to answer and fulfil the needs and requirements of four primary stakeholders: patients, healthcare providers, researchers, and policymakers. Each stakeholder would benefit from a dedicated set of data types, meticulously aligned with specific interests and objectives.

Patients benefit from BEACON as a valuable resource that systematises pertinent information on cancer-related patient associations, as well as resources available at both national and hospital level. Additionally, the project gathers data on ongoing clinical trials, providing patients interested in participating in a trial with easy-to-access information. One of the key features of BEACON’s patient-focused data is the inclusion of detailed hospital capacity information that can assist patients in making informed decisions about their cancer care needs and enhances shared decision-making, which is a significant patient preference [14]. This functionality is exemplified through a case study involving a patient navigating healthcare options for a diagnostic procedure. Using the BEACON tool, the patient identifies and compares healthcare facilities based on several factors such as hospital capacity, specialist expertise, and patient reviews. This case study underscores BEACON’s practical application in empowering patients to make informed and timely decisions, demonstrating the platform’s transformative potential in improving patient care outcomes.

Healthcare providers can access information regarding the availability of experts across different departments of cancer centres. Rather than collecting personal data on individual experts, BEACON directs users to cancer centres’ own pages, which offer comprehensive information on various specialists. Furthermore, the project maps clinical practice guidelines and learning resources from these centres, providing valuable educational materials for healthcare providers.

Regarding researchers, BEACON collects metadata on a wide range of cancer-related databases, including administrative/claims databases, cancer registries, and databases from clinical trials, surveys, and biobanks. This wealth of data empowers researchers by allowing them to access, in a centralised, easy-to-access wiki, metadata on diverse sources of information essential for their studies and searches. Moreover, BEACON includes hospital capacity information, which can assist researchers in identifying suitable healthcare facilities for conducting future clinical trials and research endeavours.

For policymakers, BEACON collects information on policy-making initiatives and reports that are crucial for informed decision-making and resource allocation strategies in cancer healthcare policy. Additionally, comparative reports on disparities in cancer care across the EU as well as reports on hospital capacities are provided by BEACON. This kind of information and service can contribute in shedding light on potential correlations between regional disparities and hospital capabilities, thereby informing policy makers on areas requiring attention and intervention [9, 15].

To address whether a consolidated oncology metadata repository can enhance data access and inform decisions in cancer care across the EU, BEACON employs a rigorous metadata retrieval methodology. This ensures that the collection, reliability, and accessibility of data sources align with the overarching aim of improving data-driven decision-making. In addition, BEACON employs various methods tailored to each data type, mainly focussing on descriptive details about data sources, data coverage, and access to protocols. Indeed, BEACON exclusively gathers metadata and avoids the collection of any patient-specific or sensitive data, thus ensuring compliance with privacy regulations and ethical standards [16].

To gather information on hospital capacities, BEACON used a multifaceted approach, including manual examination of hospital websites across the EU and semiautomated methods exploiting Application Programming Interfaces (APIs) from PubMed and ClinicalTrials.gov. Comparably, regarding databases, BEACON explored websites of data holders such as cancer registries, ministries of health, health organisations, and academic institutions. Additional information was retrieved from the literature describing these databases.

Focusing on data relevant to policymakers, reports and clinical practice guidelines were obtained from websites of national health and cancer institutions. Lastly, information on quality of life and palliative care was gathered through targeted searches on PubMed using specific Medical Subject Headings (MeSH) terms.

In order to clarify information and incentivise feedback on the content included in the Beacon Wiki, engagement with database holders, patient associations, and hospitals was managed via email. This contributed to ensuring the accuracy and completeness of the gathered data, further enhancing the reliability and utility of the Beacon project.

In terms of platform selection and customisation, BEACON prioritises a platform that supports public accessibility, structured organisation, flexibility for the implementation of new features, and collaborative community engagement. MediaWiki emerges as the optimal choice, offering an intuitive system for content creation, editing, and moderation. Lastly, to enhance user-friendliness and utility, extensions and plugins are implemented to facilitate customisation as well as international language support was prioritised to foster inclusivity and accessibility across the EU.

Overall, the Beacon project represents a significant initiative aimed at providing a valuable resource for stakeholders involved in cancer care. Through its diligent and careful data collection efforts, rigorous methodology, and user-friendly platform, BEACON strives to empower patients, healthcare providers, researchers, and policymakers with the information they need to make informed decisions and drive positive change in cancer healthcare policy and practice.

## Utility and discussion

The BEACON project, established with the ambitious objective of meticulously mapping oncological information across the 27 member states of the EU, has now achieved its overarching goals. This section outlines the utility of the BEACON database, discussing our finalised findings, key observations, and the enhancements implemented to support ongoing cancer care and policy development.

### Key findings

The main achievement of BEACON lies in establishing a rich repository of over 1,200 oncological data sources (including over 200 administrative/claims databases and cancer registries). In relation to our research question, these findings suggest that a centralised repository could indeed facilitate improved access to and understanding of oncological data. This, in turn, may support data-driven decision-making, reduce information silos, and inform targeted efforts to address cancer care disparities within the EU. Another key outcome is the Disparity Atlas, aimed at exploring the association between hospital capacity and healthcare disparities, and, thus, empower policymakers with actionable insights to guide targeted interventions and reduce disparities in cancer care. BEACON’s commitment lies in equipping stakeholders with comprehensive metadata encompassing crucial details such as data dictionaries, access protocols, and hospital capacity insights, empowering users with appropriate understanding of available resources thus facilitating utilisation. Moreover, BEACON supports advanced techniques like Resource Description Framework (RDF) tagging and data analysis to enhance the accessibility and utility of its mapped information. By embracing innovative methodologies, BEACON not only ensures the accessibility of its resources but also unlocks valuable insights for stakeholders, promoting a data-driven approach to cancer care and policy formulation [17].

### Key observations

While BEACON has made significant progress in its project duration, there are still challenges future projects could tackle. As data is widely recognised as a crucial tool for the improvement of population health, one notable challenge is the accessibility of some databases, with many lacking publicly available dictionaries or featuring dictionaries that are difficult to locate. The latter issue mirrors challenges faced by Low-and Middle-Income Countries (LMICs) in maintaining and accessing medical data archives, which represent important barriers to the development of resilient health systems. In order to maximise the utility of BEACON resources, and maximise user experience, it is crucial to address these issues [18].

Furthermore, the main focus of databases on cancer incidence and mortality data underlines a notable gap in the availability of critical information related to health determinants. Indeed, notable efforts should be directed towards increasing the scope of available data to encompass a broader spectrum of health determinants, achieve a more holistic understanding of cancer epidemiology and inform comprehensive interventions [19].

Finally, the limited engagement from database providers underscores the importance of fostering greater collaboration and communication between BEACON and data providers. By establishing robust partnerships and incentivising active participation, BEACON can enrich its repository and ensure the continuous expansion and improvement of its resources.

### Decision support system

In addition to the comprehensive Beacon Wiki repository, we have developed a dedicated decision support application aimed at improving information access and decision-making for our four primary stakeholders: patients, healthcare providers, researchers, and policymakers. Users begin by selecting a profile that tailors the information they receive, directing them to the most relevant content from the Wiki based on their specific needs. Further customization is possible through filters by information type, cancer type, and country, providing a flexible, user-friendly search experience.

To enhance accessibility and inclusivity, the application supports all 24 official EU languages and features a location-based setting that automatically adjusts the language interface. This is particularly beneficial for users who are less experienced with digital tools, ensuring that vital information is always presented in a familiar language. Initial testing of this decision support system yielded positive feedback, indicating that it successfully streamlines access to critical oncological data and improves the overall usability of the Beacon initiative for all stakeholders. Additionally, once the application was released to the public, BEACON consistently reached an average of 200 users every month, visiting a variety of BEACON pages and metadata.

### Database utility and envisioned benefits

The BEACON database holds notable potential of contribution to improving the landscape of oncological data management and utilisation. For patients, it will offer access to vital information for informed decision-making about cancer care facilities and resources. Healthcare providers will benefit from dedicated data on experts availability and clinical guidelines, empowering them to deliver tailored and evidence-based care.

Similarly, researchers can access a diverse array of oncological databases for scientific inquiry and discovery, promoting collaborations and driving advancements in cancer research. Policymakers, provided with comparative reports and actionable insights, are prompted to formulate evidence-based policies and interventions aimed at reducing disparities and improving cancer outcomes across the EU.

While existing databases may offer similar functionalities, BEACON distinguishes itself through its comprehensive metadata, extensive resource repository, and innovative methodologies. By focusing on EU-wide mapping and employing advanced techniques like RDF tagging, BEACON sets a new standard in oncological data management and analysis, set to assemble transformative change in cancer care and policy.

### *Case study*: utilising BEACON for informed decision-making

#### Scenario

Alexandra, a 56-year-old woman, recently underwent a routine health check-up and received concerning results prompting her physician to recommend further diagnostic testing. Alexandra’s physician suspects a potential gastrointestinal issue and advises her to undergo a specific diagnostic exam, known as a colonoscopy, to investigate further. Alexandra, eager to understand her options and locate a trusted healthcare facility for the procedure, turns to BEACON for guidance.

#### Process

Accessing BEACON

1. Alexandra navigates to the BEACON Wiki platform via her smartphone or computer, where she encounters a user-friendly interface designed for easy navigation and accessibility.

Search for information

2. Alexandra uses BEACON’s search functionality to find relevant information about colonoscopy facilities in her area. To refine her search, she inputs keywords such as “colonoscopy,” “gastrointestinal exam,” and her region or city.

Reviewing search results

3. BEACON returns a list of comprehensive search results, including information on various healthcare facilities offering colonoscopy services in Alexandra’s city or region. Each result includes detailed metadata, such as the facility’s accreditation status, available equipment, expertise of healthcare providers, patient reviews, and contact information.

Evaluating options

4. Alexandra carefully reviews the search results, comparing factors such as the proximity of facilities to her city or region, the reputation of healthcare providers, the availability of specialised equipment, and patient feedback. She considers additional information provided by BEACON, such as hospital capacity insights and quality of care indicators, to make an informed decision.

Selecting a healthcare facility

5. After thorough consideration, Alexandra selects a healthcare facility that meets her criteria and aligns with her preferences. She notes the contact details provided by BEACON and proceeds to schedule an appointment for her colonoscopy procedure.

#### Outcome

Alexandra’s experience with BEACON illustrates the platform’s effectiveness in facilitating informed decision-making and empowering patients to take an active role in managing their healthcare. By searching BEACON’s comprehensive database of healthcare facilities and relevant metadata, Alexandra successfully identifies a suitable facility for her colonoscopy, ensuring timely access to high-quality care. The seamless navigation, detailed information, and user-friendly interface of BEACON contribute to Alexandra’s positive experience, highlighting the practical utility and transformative potential of the database in the clinical setting.

### Limitations and sustainability

While BEACON successfully consolidates and standardises oncological metadata across the EU, several limitations warrant attention. First, despite efforts to engage all member states, varying levels of participation and data-sharing culture resulted in uneven data coverage. Some countries or institutions provided comprehensive metadata, while others offered only limited or hard-to-access information. Although the repository includes multiple data types—from hospital capacities to policy initiatives—certain granular data (e.g., socioeconomic determinants or real-time clinical outcomes) remain incomplete or unavailable.

Additionally, the initial development phase relied on the EU4Health funding mechanism. Ensuring long-term sustainability of the Beacon Wiki will require a stable governance structure, ongoing financial support, and sustained stakeholder engagement. This includes establishing partnerships with regulatory bodies, cancer research networks, and patient advocacy groups to continually update and validate the data. We developed a sustainability plan to address these challenges and ensure the Wiki and the Decision Support Application’s long-term viability.

Finally, while the wiki-based approach encourages user contributions and transparency, it also relies on active community participation for content accuracy and currency. We have implemented clear editorial guidelines, quality control measures, and moderator oversight to mitigate the risk of misinformation or content stagnation.

By acknowledging these limitations and implementing strategies to address them, we expect to enhance the platform’s reliability, longevity, and overall value to the European oncology community.

## Conclusions

In conclusion, the BEACON project has demonstrated how a centralised, open-access repository of oncological data can improve the accessibility and relevance of information for patients, healthcare providers, researchers, and policymakers across the European Union. By providing a structured, user-friendly platform that integrates multiple languages and tailoring content to individual users’ needs, BEACON supports more informed decisions and the development of data-driven approaches to cancer care. Despite significant challenges, BEACON has demonstrably improved the accessibility and utility of oncological data across the EU. By fostering collaboration, prioritising equity, and embracing innovation, the repository supports a more informed and cohesive oncology community. Such transparency and engagement ultimately serve not only to guide policy and clinical practice but also to encourage continuous improvement, thereby enhancing long-term sustainability.

## Electronic supplementary material

Below is the link to the electronic supplementary material.

Supplementary Material 1

## Data Availability

The datasets generated and/or analysed during the current study are available in the BEACON Wiki repository: https://wiki.beaconcancer.org/index.php?title=Cancer_Beacon.

## Abbreviations

EU: European Union
US: United States
EHDs: Electronic Health Data
GDPR: General Data Protection Regulation
IARC: International Agency for Research on Cancer
OECD: Organisation for Economic Co-operation and Development’s
BEACON: Cancer Care Beacon Project
APIs: Application Programming Interfaces
MeSH: Medical Subject Headings
RDF: Resource Description Framework
LMICs: Low-and Middle-Income Countries
