# Supplementary material for: The Beacon Wiki: Mapping oncological information across the European Union
Source: BMC Med Inform Decis Mak. 2025 May 19;25:193. doi: 10.1186/s12911-025-03015-6 (PMC12090651; doi:10.1186/s12911-025-03015-6)
Supplement: Supplementary file 1 — Supplementary Material 1 [file 12911_2025_3015_MOESM1_ESM.pdf]

Supplementary Material

A. BEACON Metadata Retrieval Methodology

The strategy adopted for metadata collection was multi-pronged and adapted to the nature of each information category. For cancer-related databases—including administrative and claims data, cancer registries, clinical trial datasets, surveys, and biobanks—we conducted systematic web searches for each EU member state. We investigated websites belonging to cancer registries, ministries of health, public health institutes, universities, and national statistics agencies. Each relevant source was examined to identify the existence of databases and to extract available metadata such as the data holder, primary purpose, cancer areas covered, data types, temporal coverage, access protocols, and associated documentation. In addition to these manual searches, we applied semi-automated methods to retrieve literature references that described or referenced such databases. These publications were used as secondary sources when direct access to the metadata was not possible.

To support decision-making for policymakers and providers, we collected national and institutional reports as well as clinical practice guidelines. These were identified through manual searches of official websites maintained by national health agencies, cancer institutions, and international health organisations. We focused on documents related to cancer screening, diagnosis, treatment pathways, and palliative care strategies. Each item was evaluated and documented based on its scope, authorship, relevance, and usability within the BEACON framework.

For hospital capacity information, we employed a combination of methods. A manual review of hospital websites provided data on departments, oncology services, and facility resources. To supplement this, we used application programming interfaces (APIs) from PubMed and ClinicalTrials.gov to gather information about the hospital's involvement in cancer-related research and care delivery. This approach allowed us to better assess each hospital's capacity in areas such as screening, diagnosis, treatment, survivorship, and palliative care.

At both the national and institutional levels, we compiled directories of cancer-related patient associations and support resources. This effort involved reviewing official health portals, non-profit registries, and hospital-affiliated outreach programs. When available, we included information such as the association's objectives, the types of cancer they focus on, and the nature of the support they offer to patients. For cancer centers specifically, we created pages that highlight ongoing clinical trials, departmental expertise (by linking to staff directories without including personal details), available learning materials for both patients and professionals, and patient support services offered at the institutional level.

Throughout the process, we also engaged in direct outreach by email to institutions, database custodians, hospitals, and patient organisations. These communications were aimed at verifying the accuracy of the collected data, requesting clarification when information was ambiguous or incomplete, and inviting feedback on content already included in the BEACON Wiki. The response rate and the level of engagement from these stakeholders are analysed in the next section of this report.

B. Sustainability Plan

The long-term sustainability of the BEACON Wiki and its accompanying Decision Support Application will be achieved through strategic alignment with clinical, technological, and policy-oriented initiatives that ensure continued relevance and integration into the European oncology landscape. Central to this sustainability plan is the use of BEACON hospital capacity data to support the operational phases of clinical trials. By enabling the identification of hospital catchment areas and infrastructural resources, BEACON can directly inform trial feasibility assessments and recruitment strategies. This functionality allows the platform to serve as a dynamic resource in clinical research planning, thereby embedding it into the ongoing infrastructure of trial design and coordination across EU member states.

Another key element of the sustainability strategy is the integration of BEACON metadata into large

language models used for patient-centered clinical decision support. By incorporating location-specific hospital and service information into these AI systems, BEACON enhances the personalization and contextual relevance of recommendations offered to patients and clinicians. This approach not only improves individualized care but also ensures that the BEACON platform remains actively utilized and updated within emerging digital health tools.

Additionally, BEACON serves as a foundational resource for investigating disparities in cancer care across Europe. The structured metadata on hospital capacities and regional services supports analyses that explore the association between healthcare infrastructure and outcome inequalities. This functionality aligns the platform with public health and policy efforts aimed at addressing systemic inequities in cancer diagnosis, treatment, and survivorship. As research and policy efforts increasingly emphasize data-driven strategies for advancing health equity, BEACON is positioned as a critical resource for sustained academic and institutional engagement.
